# Formaldehyde at Low Concentration Induces Protein Tau into Globular Amyloid-Like Aggregates *In Vitro* and *In Vivo*

**DOI:** 10.1371/journal.pone.0000629

**Published:** 2007-07-18

**Authors:** Chun Lai Nie, Yan Wei, Xinyong Chen, Yan Ying Liu, Wen Dui, Ying Liu, Martyn C. Davies, Saul J.B. Tendler, Rong Giao He

**Affiliations:** 1 State Key Laboratory of Brain and Cognitive Science, Institute of Biophysics, Graduate School, Chinese Academy of Sciences, Chaoyang District, Beijing, China; 2 Laboratory of Biophysics and Surface Analysis, School of Pharmacy, The University of Nottingham, Nottingham, United Kingdom; Baylor College of Medicine, United States of America

## Abstract

Recent studies have shown that neurodegeneration is closely related to misfolding and aggregation of neuronal tau. Our previous results show that neuronal tau aggregates in formaldehyde solution and that aggregated tau induces apoptosis of SH-SY5Y and hippocampal cells. In the present study, based on atomic force microscopy (AFM) observation, we have found that formaldehyde at low concentrations induces tau polymerization whilst acetaldehyde does not. Neuronal tau misfolds and aggregates into globular-like polymers in 0.01–0.1% formaldehyde solutions. Apart from globular-like aggregation, no fibril-like polymerization was observed when the protein was incubated with formaldehyde for 15 days. SDS-PAGE results also exhibit tau polymerizing in the presence of formaldehyde. Under the same experimental conditions, polymerization of bovine serum albumin (BSA) or α-synuclein was not markedly detected. Kinetic study shows that tau significantly misfolds and polymerizes in 60 minutes in 0.1% formaldehyde solution. However, presence of 10% methanol prevents protein tau from polymerization. This suggests that formaldehyde polymerization is involved in tau aggregation. Such aggregation process is probably linked to the tau's special “worm-like” structure, which leaves the ε-amino groups of Lys and thiol groups of Cys exposed to the exterior. Such a structure can easily bond to formaldehyde molecules *in vitro* and *in vivo*. Polymerizing of formaldehyde itself results in aggregation of protein tau. Immunocytochemistry and thioflavin S staining of both endogenous and exogenous tau in the presence of formaldehyde at low concentrations in the cell culture have shown that formaldehyde can induce tau into amyloid-like aggregates *in vivo* during apoptosis. The significant protein tau aggregation induced by formaldehyde and the severe toxicity of the aggregated tau to neural cells may suggest that toxicity of methanol and formaldehyde ingestion is related to tau misfolding and aggregation.

## Introduction

Neuronal tau is an important protein in promoting and stabilizing the microtubule system involved in cellular transport and neuronal morphogenesis. The tau molecule can be subdivided into an amino-terminal domain that projects from the microtubule surface and a carboxy-terminal microtubule-binding domain. The discovery that incubation of bacterially expressed human tau with sulphated glycosaminoglycans leads to bulk assembly of tau filaments [1], making it possible to obtain structural information [2]. By using circular dichroism measurement, Schweer *et al*. have found that protein tau lacks secondary structures and is considered in a “worm-like” conformation with a high flexibility [3]. Therefore, the side-chains of amino acids such as Lys, Cys, Thr and Ser are mostly exposed and vulnerable to chemical modification. Recently, many laboratories have found that misfolding and aggregation of protein tau are involved in neurodegeneration [2], [4]–[6]. Protein tau has been found as the major component of paired helical filaments in neurofibrillary tangles in the brains of Alzheimer's patients, where abnormal hyper-phosphorylation induces tau to misfold and form the paired helical filaments, depositing in the cytoplasm of neurons [7]–[10].

Recently, a great deal of evidence has demonstrated that oxidation and glycation stresses are key causal factors of neuronal degenerative diseases [11]–[13]. Both of them inevitably produce a variety of unsaturated carbonyls as intermediates, like malondialdehyde and 4-hydroxynonenal, which usually cause carbonyl-amino crosslinking and lead to accumulation of irreversible changes (like lipofuscin) related to various neurodegenerative diseases in particular [14]–[16]. Such carbonyl stress-related reactions (carbonylation) can form unstable and reversible 1∶1 amino-carbonyl (Shiff's base) compounds at an early stage of protein modification [16], [17]. Carbonylation binds and blocks α-/ε- amino groups, and results in changes in charge and conformation of a protein. In order to investigate the relationship between carbonylation and protein tau misfolding, the basic and simplest carbonyl compound formaldehyde [18] has come into our attentions.

Formaldehyde is a common environmental agent found in paint, cloth, exhaust gas and many other medicinal and industrial products [19]. Formaldehyde exposure leads to formation of DNA/protein crosslinks, a major mechanism of DNA damage. The DNA/protein crosslinks have been used as a measure of dose in drug delivery [20]. Formaldehyde, as a crosslinking agent, also reacts with thiol and amino groups, leading to protein polymerization [21], [22]. Furthermore, methanol ingestion is an important public health concern because of the selective actions of its toxic metabolites, formaldehyde and formic acid, on the retina, the optic nerves and the central nervous system (CNS) [23]. Illicit consumption of industrial methylated spirits can cause severe and even fatal illness [24]. In the liver and retina, methanol is oxidized by alcohol dehydrogenase, resulting in formaldehyde. In semicarbazide-sensitive amine oxidase (SSAO)-mediated pathogenesis of Alzheimer's disease, formaldehyde interacts with β-amyloids and produces irreversibly cross-linked neurotoxic amyloid-like complexes [21], [22], [25].

We have examined the role of formaldehyde in misfolding of protein tau [26]. In particular, we investigated the toxicity of formaldehyde-induced tau aggregates on human neuroblastoma cells (SH-SY5Y cell line) and rat hippocampal cells [27]. The results showed that low concentrations (0.01–0.1%) of formaldehyde are sufficient to induce formation of amyloid-like tau aggregates, which can induce apoptosis of both SH-SY5Y and hippocampal cells. This may be significant to understand the mechanism of chronic damage caused by methanol toxicity and formaldehyde stress [18], [28]. However, we have still not known the mechanism of protein tau aggregation in the presence of formaldehyde at low concentrations. The present study concerns the characteristic of misfolding and polymerization of extracellular and intracellular neuronal tau induced by formaldehyde at low concentrations.

## Results

### Changes in size of tau aggregates at different concentrations of formaldehyde

**Figure 1:**
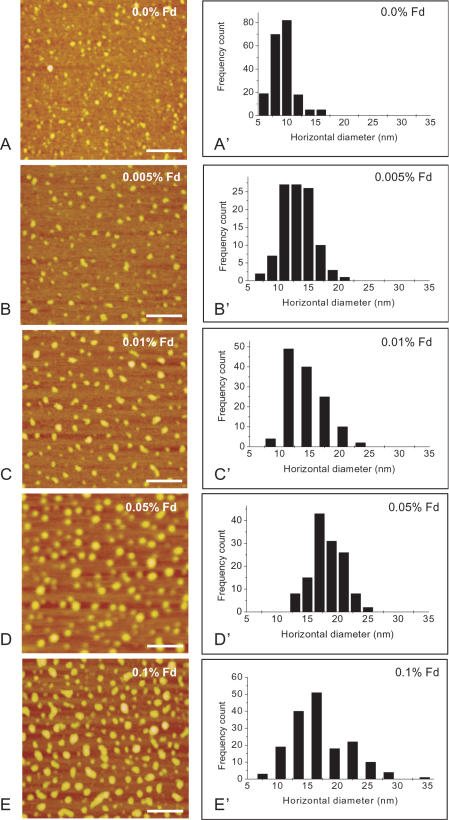
AFM Images of neuronal tau in the presence of formaldehyde at different concentrations. Neuronal tau (20 µM) was incubated in 50 mM phosphate buffer (pH 7.2) containing formaldehyde at different concentrations as indicated at 37°C over night (A–E). Aliquots were taken and diluted to the desired concentration using the phosphate buffer, and the samples were dropped onto mica surfaces and dried in air before observed under the atomic force microscope. The frequency counts of the horizontal diameters (explained in the text) of the protein particles in images A–E were shown in A'–E', respectively. The scale bars equal 100 nm.

**Figure 2:**
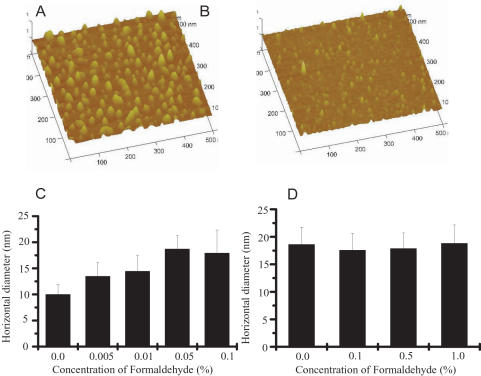
Three-dimensional images and horizontal diameters of tau at different concentrations of formaldehyde. The data using for regenerating three-dimensional images of neuronal tau in the presence (A) and absence (B) of formaldehyde solution (0.1%) are the same used for Figures 1A and E. Change in the horizontal diameter of the tau protein particles on mica surface at different concentrations of formaldehyde is depicted in C. Change in the horizontal diameter of BSA was used as control (D).

AFM observation of native and formaldehyde-treated neuronal tau is presented in Figure 1. All images were obtained in air. Both native and formaldehyde-treated tau appeared as globular particles but of different sizes. Native tau showed globules with diameter of approximately 9±2 nm (Figure 1A). The diameter increased significantly in the presence of formaldehyde, to 18±3 nm when tau was incubated in the presence of only 0.05% formaldehyde (Figure 1D), about twice of that of native tau. The diameter increased following the increase of formaldehyde concentration (Figure 1B–E). Histograms presented in Figures 1A' through to E' showed particle diameter distributions in the presence of formaldehyde at different concentrations. The data indicate that the presence of formaldehyde, even at very low concentrations, results in polymerization of protein tau. For a better visual effect to show the particle size difference, the AFM images of native and 0.1% formaldehyde-treated tau were also presented in a “three-dimensional” fashion in Figure 2A and B. It is also worthwhile to mention that no fibrils were ever observed in spite of the particle size increase in the presence of formaldehyde.

Though the polymerization of tau was easily observed in the presence of formaldehyde (Figure 2C), as a control, no significant polymerization of BSA was observed under the same conditions (Figure 2D). This suggests that the conformation of neuronal tau is more sensitive to formaldehyde than that of BSA.

To control the AFM observation, neuronal tau incubated with different concentrations of formaldehyde was electrophoresed on SDS-PAGE (Figure S1A). Existence of tau polymers was detected on the PAGE following the increase of formaldehyde concentration. Measurement of gray density of the protein bands on the SDS-PAGE (Figure S1B) exhibits a noticeable increase of tau polymers and a corresponding decrease of tau monomers in the same range of formaldehyde concentration as observed in AFM experiments.

Acetaldehyde was used as a negative control. No tau polymer was detected by SDS-PAGE when acetaldehyde, instead of formaldehyde, was used under the same conditions (Figure S1C and S1D). This result was further supported by AFM observation, where a constant particle diameter of about 10 nm was observed when acetaldehyde concentration was changed from 0.1% to 1% under the same conditions (Figure S2).

Glutaraldehyde (0.01–1%) was used as a positive control. The AFM images were not shown but the mean values of particle diameters at different glutaraldehyde concentrations are presented in Figure S3. The results showed that both neuronal tau and BSA were polymerized in the presence of glutaraldehyde under the same conditions. The diameters of observed particles for tau and BSA were 9.2±1.2 nm and 17.5±2.4 nm, respectively, in the absence of glutaraldehyde whilst 21.83±2.67 nm and 37.22±3.57 nm, respectively, in the presence of glutaraldehyde.

### Kinetic course of tau aggregation in formaldehyde solution

**Figure 3:**
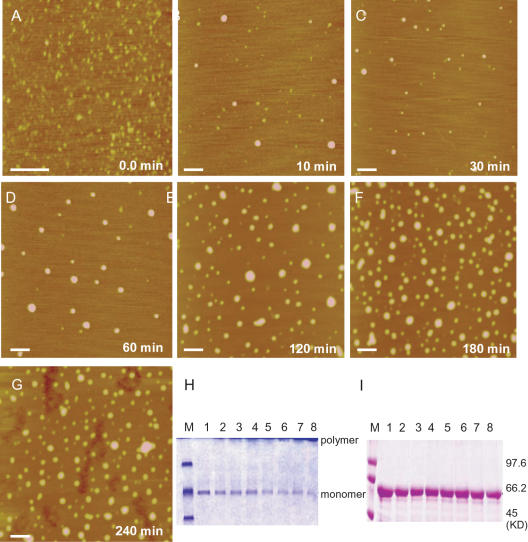
Kinetic aggregation of tau in formaldehyde solutions. Neuronal tau was added to the phosphate buffer (pH 7.2, 37°C) containing 0.1% formaldehyde, and aliquots were taken for observation under the atomic force microscope at different time intervals as indicated in each images (A–G). The scale bars equal 100 nm. The parallel SDS-PAGE study (H) shows tau incubated with 0.1% formaldehyde for different time periods, with Lanes 1 to 8 representing 0, 30, 60, 90, 120, 150, 180 and 240 min, respectively. Lane M is the protein molecular mass marker. Acetaldehyde (0.1%) was used as a control and the result is shown in I.

**Figure 4:**
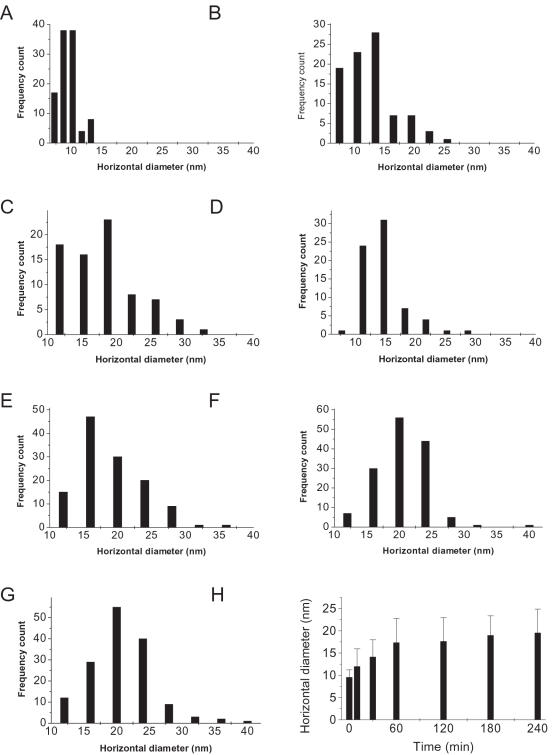
Changes in horizontal diameters of tau at different time intervals in 0.1% formaldehyde solution. The frequency counts of the horizontal diameters of tau protein at different incubation time (A–G) are based on the AFM images shown in Figures 3A–G, respectively. The mean values are summarized in H.

To investigate the kinetics of tau aggregation in the presence of low-concentration formaldehyde, neuronal protein tau was incubated in 0.1% formaldehyde and aliquots were taken for AFM observation at different time intervals. The AFM images were shown in Figure 3A–G whilst the corresponding particle diameter distributions were shown in Figure 4A–G. The results showed a rapid start (within 10 min) of aggregation in the presence of formaldehyde and a remarkable increase of particle size in about 60–120 min. Parallel SDS-PAGE measurements also revealed a similar time evolution (Figure 3H). The kinetic change in the size of aggregates showed a rapid polymerization of tau during the first 60 min (Figure 4H). Analysis of the polymerizing kinetics with Tsou's method [29] showed a linear procedure for tau polymerization in the presence of formaldehyde with a constant of the first order rate of 2.6×10^−4^ s^−1^. As a control, no aggregation was detected by SDS-PAGE in the same time course when acetaldehyde, instead of formaldehyde, was added to the tau solution (Figure 3I).

**Figure 5:**
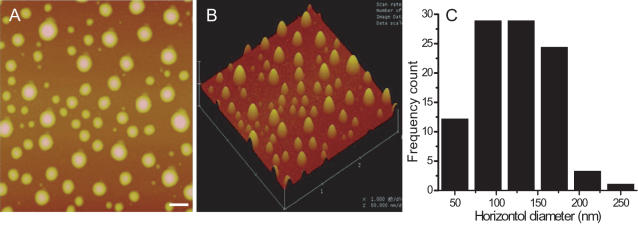
Aggregates of tau incubated in 0.1% formaldehyde for 15 days. The experimental conditions were described in Figure 3, except that the incubation time was prolonged to 15 days. A typical AFM image of tau in the presence of formaldehyde is present in A (Bar: 100 nm), together with a three dimensional image (B) for a better visual effect. Frequency counts of horizontal diameters of particles are presented in C.

Furthermore, to confirm that formaldehyde induces tau into globular aggregates, we had incubated protein tau with 0.1% formaldehyde for 15 days, and then observed the aggregation under AFM. As shown in Figure 5, the polymer of tau was still in globular aggregation, and no fibrils could be found under the experimental conditions.

**Figure 6:**
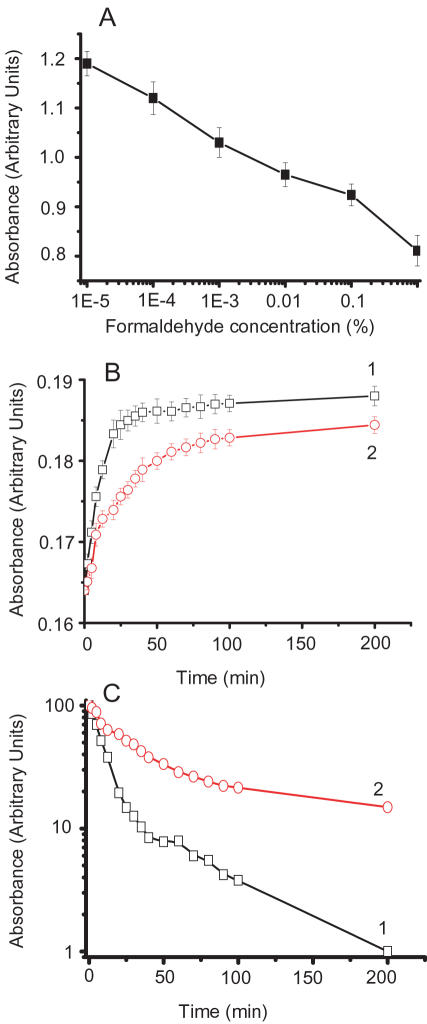
Reaction of formaldehyde with thiol groups of neuronal tau. Neuronal tau (2 µM) was re-suspended in phosphate buffer containing DTNB (20 times in excess to the protein in molar ratio) in the presence of formaldehyde at different concentrations at 37°C for 120 min. The DTNB absorbance (A, 412 nm) was measured. Under the same conditions, native tau (Curve 1) and 0.005% formaldehyde-treated tau (Curve 2) were re-suspended in 2 µM DTNB (B), and aliquots were taken to measure the absorbance at different time intervals. The same data shown in B are also plotted in semilogarithm (C) according to Tsou's method [29].

It is known that formaldehyde reacts with the side chain of amino acid residues containing ε-amino and thiol groups [21]. Tau-40 contains 44 lysine and 2 cysteine residues (NP_005901) [1], [30], which should react with formaldehyde. 5,5′-Dithio-bis(2-nitrobenzoic acid) (DTNB) modification was used to check the thiol groups of tau incubated under different concentrations of formaldehyde (Figure 6) whilst *o*-phthaldehyde (OPT) modification was used to detect the amino groups of tau under the same conditions as described previously [27]. Both DTNB absorbance (412 nm) and OPT fluorescence (E_m_ 455 nm/E_x_ 340 nm) decreased following the increase of formaldehyde concentration. In kinetic study, as shown in Table 1, the first order rate constants of DTNB and OPT reacting with formaldehyde-treated tau were much lower than those with native tau. This indicates that both thiol and amino groups of tau were blocked in the presence of formaldehyde.

**Table 1:** The first order rate constants of DTNB and OPT reacting with native tau and 0.005% formaldehyde-treated tau.

	In the presence of DTNB		In the presence of OPT	
	Native tau	Formaldehyde-treated tau	Native tau	Formaldehyde-treated tau
Fast phase	130.2±7.01	43.5±3.01	81.3±6.13	7.7±0.91
Slow phase	23.4±3.42	5.6±0.87	32.1±2.05	7.4±0.79

Data are in 10^5^ s^−1^.

**Figure 7:**
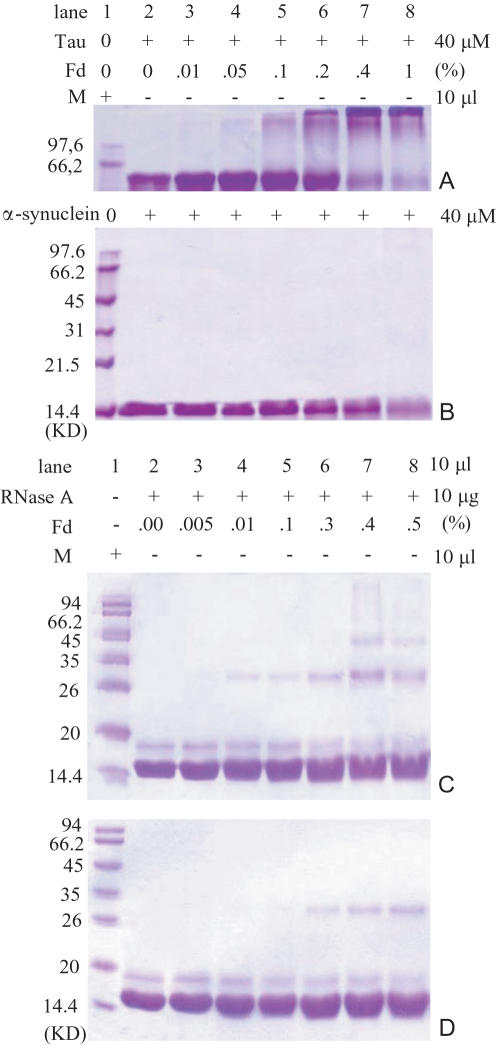
Formaldehyde-treated α-synuclein and RNase A on SDS-PAGE. Protein tau (A) or α-synuclein (amino acid sequence referred to NP_000336) (B) was incubated in 100 mM phosphate buffer (pH 7.2) containing formaldehyde at different concentrations as indicated at 37°C over night, and then aliquots were taken for SDS-PAGE. RNase A (3 mg/ml) was pretreated with DTT (10 mM) in 100 mM phosphate buffer (pH 7.2) at 37°C for 1 h and dialysed at 4°C over night as described [31]. The dialysed RNase A (10 µg) was incubated with formaldehyde at different concentrations as indicated at 37°C for 4 h (C). Aliquots were then taken for SDS-PAGE. RNase A incubated without DTT was used as control (D).

To confirm the contribution of thiol groups to tau aggregation, α-synuclein, which does not contain any thiol group (NP_000336), was employed in this work. As shown in Figure 7B, polymerizaion of α-synuclein could hardly be observed in SDS-PAGE when formaldehyde concentration was less than 1% (though the monomer slightly decreased in the solutions). This indicates that α-synuclein is less vulnerable to the induction of the aldehyde than protein tau (Figure 7A). On the other hand, ribonuclease A (RNase A) contains 4 disulfide bridges which can be reduced by dithiothreitol (DTT) [31]. The oligomer of reduced RNase A increased observably in the presence of 0.01% formaldehyde (Figure 7C). The intact RNase A, however, started to aggregate when formaldehyde concentration increased to 0.3% (Figure 7D) under the same conditions.

### Formaldehyde self-polymerization and tau aggregation

**Figure 8:**
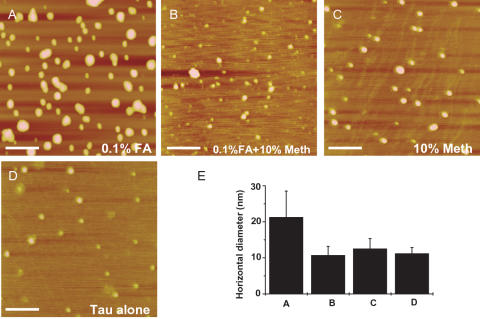
Effect of 10% methanol on neuronal tau aggregation. Conditions were referred to Figure 1, except that additional methanol was used to disturb the reaction of formaldehyde with neuronal tau. The images show neuronal tau incubated in phosphate buffer in the presence of 0.1% formaldehyde only (A); mixture of 10% methanol and 0.1% formaldehyde (B); 10% methanol only (C); and tau alone (D). The scale bars equal 100 nm. The corresponding horizontal diameters are summarized in E.

According to Pomerantz *et al*. [32], formaldehyde polymerizes itself in water whilst 10–15% methanol can prevent such polymerization. To investigate the methanol effect, we did AFM experiments of tau with existence of 0.1% formaldehyde (Figure 8A), 10% methanol (Figure 8C) and mixture of 0.1% formaldehyde with 10% methanol (Figure 8B), in comparison with the native tau (Figure 8D). The mean values of observed particle diameters in the four cases are illustrated in Figure 8E, which showed that as long as methanol is present (Figure 8B and C), the particle sizes were similar to that of native tau (about 10 nm in diameter). This indicates that no aggregation occurs in the presence of methanol, even with existence of formaldehyde. Only in the presence of formaldehyde alone, aggregation with a diameter of about 22±7 nm was observed. This suggests that formaldehyde self-polymerization plays an important role in tau aggregation and polymerization. If formaldehyde polymerization was blocked, as in the present case, by methanol, no further aggregation of tau would occur.

### Low concentration of formaldehyde inducing endogenous tau to aggregate

**Figure 9:**
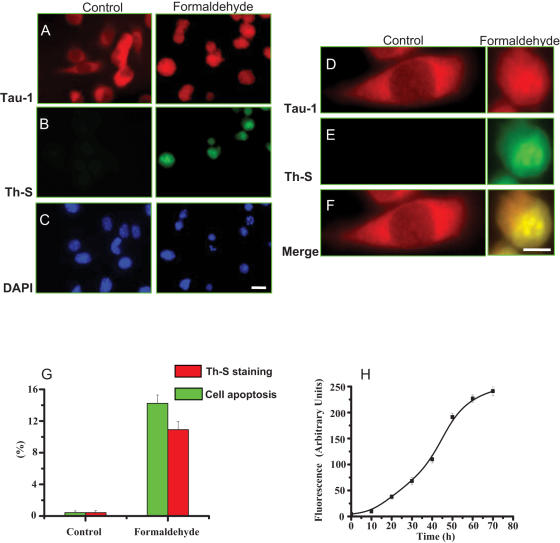
Tau aggregation in SY5Y cells in the presence of formaldehyde. SY5Y cells were cultured and incubated with 0.0005% formaldehyde for 72 h. Immunolabelling with the monoclonal antibody Tau-1 was used to visualize tau expression (A) and thioflavin S was to detect tau aggregates in the cells (B). Cells stained with DAPI showed apoptotic cells with the shrunk nuclei (C). Bar: 50 µm. D–F (magnified from A and B, Bar: 25 µm) show that the signals of ThS (amyloid-like aggregates) and the monoclonal antibody Tau-1 overlap each other in the cells. Quantitative results of protein tau aggregation and cell apoptosis are shown in G. Fluorescence intensity of tau aggregation stained by ThS during time course is shown in H.

Our previous results showed that extracellular tau aggregates were toxic to neuronal cells [27]. We detected intracellular tau aggregation by formaldehyde in cell apoptosis. According to Khlistunova *et al.* [33], tau expression was judged by fluorescence microscopy with monoclonal antibody Tau-1. In the present study, we probed the same cells using a dye thioflavin S (ThS), which characteristically fluoresced when amyloid structures with a high content of cross-β-structures were formed [34]. ThS fluorescence is a faithful marker of the aggregation of tau and its derivatives *in vitro*. Formaldehyde-treated cells displayed a strong reaction with ThS, while untreated cells did not show any signals in such a reaction. This indicates that formaldehyde induces amyloid-like aggregates in the cells. Furthermore, an overlapping of both Tau-1 and ThS signals in the cytoplasm suggests that protein tau is induced to form amyloid-like aggregates in the presence of formaldehyde (Figure 9). Importantly, tau aggregation preceded nuclear condensation and fragmentation after incubation in the presence of 0.0005% formaldehyde for 3 days. This shows that formaldehyde could induce intracellular tau to aggregate.

**Figure 10:**
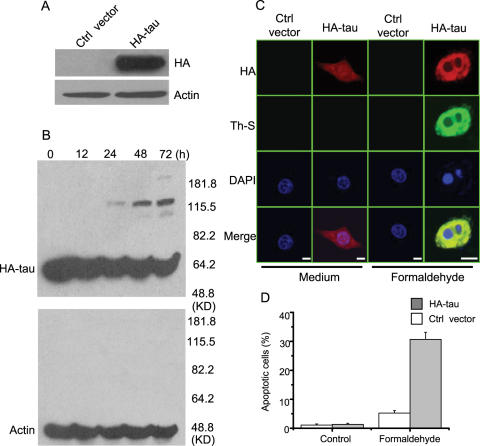
Toxicity of tau aggregation to HEK 293 cells in the presence of formaldehyde. HEK 293 cells were cultured and transient with HA-tau or the control vector, followed by incubation with 0.0002% formaldehyde for 72 h. Immunolabelling with the monoclonal antibody HA was used to visualize tau expression (A) and tau aggregation in the presence of 0.0002% formaldehyde for different times (B). β-actin was used as a protein loading control. Immunostaining detected tau aggregation in 293 cells after treated with formaldehyde for 72 h (C, Bar: 25 µm). Other conditions were similar to SY5Y cells shown in Figure 9. Quantitative analysis is presented in D. Apoptotic cells are characterized by nuclear condensation and diffraction. At least 200 apoptotic cells were counted in each experiment.

To demonstrate tau is aggregated in a cell and such aggregation is related to apoptosis, we transfected HA-tau into HEK 293 cells (Figure 10A). Note that the HEK 293 cells do not express protein tau [35]. After treated with 0.0002% formaldehyde for 72 h, tau aggregation was detectable in the transfected 293 cells by westernblotting (Figure 10B). Immunostaining remarkably visualized amyloid-like tau aggregation in the cells (Figure 10C). At the same time, apoptosis of 293-tau cells was much more obvious than that of the control cells (Figure 10D). The cells transfected with control vectors showed no aggregates and apoptosis. This suggests that formaldehyde at low concentration induces amyloid-like tau aggregates in the cells, which are related to apoptosis.

**Figure 11:**
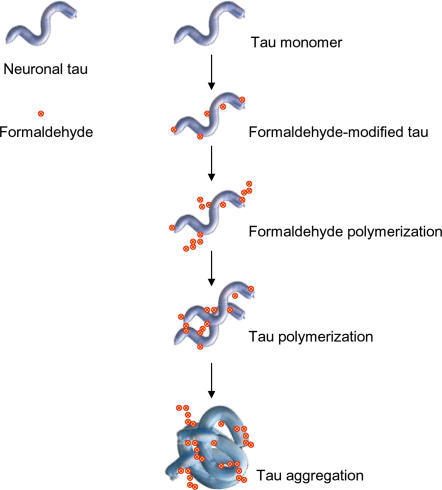
A putative mechanism for protein tau to aggregate in a formaldehyde solution. Formaldehyde reacts with the amino and thiol groups, and then formaldehyde polymerizes and induces tau to aggregate.

In conclusion, our data are well consistent with a mechanism in which formaldehyde first reacts with the thiol and amino groups of tau, and then self-polymerizes to integrate tau proteins to form the amyloid-like aggregates (Figure 11). Conceptually, formaldehyde behaves like a crosslinker to bond the formaldehyde-modified tau molecules *in vitro* or *in vivo*.

## Discussion

### Clinical lethal dose of formaldehyde

Why did we investigate tau misfolding in the presence of formaldehyde at low concentrations (0.01–0.1%)? Methanol and ethanol are metabolized to formaldehyde and acetaldehyde respectively in our hepatocytes and some neural cells [36], [37]. Both formaldehyde and acetaldehyde can go through the blood-brain barrier and cause some lesions to CNS, especially our visual system [38]. Clinically, the lethal dose of formaldehyde for human beings is about 0.08% in the circulation [39]. We have shown in the present study that formaldehyde can significantly induce tau aggregation and polymerization at concentrations even lower than 0.08%, the clinical dose of toxicosis. The same low concentration of formaldehyde did not induce polymerization of BSA though theoretically it will cause any protein to polymerize if the concentration is high enough. On the other hand, although it is known that acetaldehyde is acutely toxic and would covalently bind to proteins and other macromolecules [40], in our AFM and SDS-PAGE studies we did not observe tau polymerization caused by acetaldehyde at the concentration range that we studied (0.1–1%).

### Measurement of tau aggregate size in AFM observation

In the presence of formaldehyde at low concentrations (0.01–0.1%), globular aggregates of neuronal tau were observed in AFM when the samples were deposited on mica surfaces and dried in air, indicated by an increase of the lateral dimension of the globular particles following the increase of formaldehyde concentrations. Usually, both lateral dimension and height should be considered in particle size analysis. In the present work, we used the lateral dimension, and furthermore the “horizontal diameter”, to assess the protein polymerization. This is based on consideration of the molecular conformation. Neuronal tau is in a worm-like conformation, a native denatured state. Thus when polymerized and attached to the mica surface, the change in height may not be significant whilst the change in lateral dimension will be relatively easier to measure. To quantify the lateral dimension, we follow the statistical “horizontal diameter” method [41], [42]. In this method, the diameters of particles along each of the horizontal scan lines (or the “fast scan axis”, as called in the NanoScope AFM) are measured. All particles in the image are measured without any discrimination. In such a way, the statistic result of the horizontal diameter will provide a proper measurement of the average lateral size. Another advantage of this method is that it can avoid errors in lateral dimension measurement caused by possible image distortion caused by thermal drift, a typical instrumental artifact in scanning probe microscopy. To further guarantee the credibility, for each formaldehyde concentration that we studied, several images were used so that at least 100 particles in total were measured for each concentration.

It should be noticed that the AFM tip-broadening effect was not considered in our measurement. So, the horizontal diameter values might be overestimated. However, the most important thing is to guarantee the broadening effect is constant for comparison study. For such consideration, we always used the images obtained with the same AFM tip for quantitative analysis whenever a comparison was required.

### A putative mechanism of tau aggregation in formaldehyde solution

Formaldehyde reacts with α-/ε-amino groups and thiol groups. On the other hand, formaldehyde polymerizes itself in water [32], and the experiment shows that 10–15% methanol prevents tau polymerization. This suggests that tau aggregation may be due to the formaldehyde polymerization, which causes cross-linking between tau protein molecules. As shown in Figure 11, a tau monomer first reacts with a formaldehyde molecule, followed by more formaldehyde molecules polymerized and assembled onto the protein molecules. Finally, tau molecules are cross-linked by polymerized formaldehyde. Such a link could not be separated even on SDS-PAGE (Figure S1A and Figures 3H and 7A). However, the mechanism of tau aggregation *in vivo* needs to be further investigated.

### Difference between tau and control proteins in reaction to the presence of crosslinkers

Our present study showed that tau aggregated in the presence of low concentration formaldehyde whilst BSA and α-synuclein did not markedly. According to the previous studies [3], the conformation of native tau features a “worm-like” or a “denatured-like” structure, leaving ε-amino groups of Lys and thiol groups of Cys exposed to the exterior of the tau molecule. Thus, formaldehyde can easily bind with these side groups. For crosslinking of globular proteins, formaldehyde is not as efficient as the commonly used glutaraldehyde. Glutaraldehyde is capable of directly cross-linking two protein molecules and binds the bilateral amino groups. On the other hand, BSA is a well-folded globular protein with limited ε-amino groups exposed, and hence relatively stable under low concentration of formaldehyde. Although α-synuclein is unstructured and has the propensity to form amyloid-like aggregates [43], this protein does not contain any Cys residues. The lack of Cys residue may be a reason for α-synuclein to show a little aggregation in the presence of formaldehyde (1%). Furthermore, RNase A is a single chain polypeptide containing 4 disulfide bridges [44]. Previous gel electrophoresis study has showed that formaldehyde treatment of RNase A leads to a rapid formation of protein cross-links [45], [46]. Thus, RNase A was used as a positive control in our experiments. The DTT-treated RNase A is prone to form aggregation (Figure 7C and D). This indicates that thiol groups are involved in protein polymerization under the induction of formaldehyde. Protein tau consists of two thiol groups, Cys-291 and Cys-322 (NP_005901). The fact that neuronal tau is prone to aggregate at low concentration of formaldehyde probably reflects the special characteristics of its native conformation.

It is necessary to indicate that all proteins should be aggregated in the presence of formaldehyde if the aldehyde concentration is high enough. Different proteins require different minimum formaldehyde concentrations to induce aggregation. In comparison, protein tau is much more vulnerable to the induction of formaldehyde.

### Tau aggregation relating to methanol and formaldehyde toxicity

Methanol is an ocular toxicant, which causes visual dysfunction and often leads to blindness after acute exposure. However, physiological and biochemical changes responsible for the toxicity have not yet been well understood [28]. According to a recent report, humans are uniquely sensitive to the toxicity of methanol, as they have limited capacity to oxidize and detoxify formic acid. Thus, the toxicity of methanol in humans is characterized by formic acidaemia, metabolic acidosis, blindness or serious visual impairment, mild central nervous system depression and even death [23], [27], [28]. However, methanol toxicosis induces progressive complications to CNS. It is hard to explain the progressively chronic damage by local accumulation of formic acid alone. Therefore, the potential effect of formaldehyde on protein misfolding may be significant, although formaldehyde remains in the human body for only a short time. In semicarbazide-sensitive amine oxidase (SSAO)-mediate pathogenesis of Alzheimer's disease, formaldehyde interacts with β-amyloids and produces irreversibly cross-linked neurotoxic amyloid-like complexes [21], [22], [25]. Our studies showed that formaldehyde induced neuronal tau to aggregate. The amyloid-like tau induces apoptosis of SY5Y and hippocampal cells [27]. In fact, chemically, formaldehyde reacts with thiol and amino groups instantly, resulting in subsequent misfolding of neuronal tau (Figure 11). This suggests that amyloid-like tau is involved in methanol toxicosis, especially the damage of neurons and the resulted complications after exposure to formaldehyde.

Although there have been many studies on methanol and formaldehyde intoxication [23], [24], none of them has addressed the contribution of protein misfolding to the pathological mechanism, in particular the effect of formaldehyde on protein conformation and polymerization. Interestingly, neurofibrillary tangles have been found in brains of chronic alcoholics possessing neuropathological signs of thiamine-deficiency [40], [47]. This suggests that tau misfolding may be involved in the alcohol-induced pathological pathway. Khlistunova and his colleagues found that neuronal tau repeat domain could aggregate *in vivo* and was toxic to neuronal cells. The degree of tau aggregation and toxicity depends on the propensity of the β-structure [2], [48]. In the present study, we have demonstrated that amyloid-like intracellular tau aggregates could induce cell apoptosis, a similar result as that obtained for extracellular amyloid or α-synuclein [49]–[51]. This suggests that an enriched β-sheet structure is important to amyloid-like protein aggregation and neurotoxicity. In our experiments, a low concentration of formaldehyde induced both extracellular and intracellular tau proteins to aggregate into cell-toxic amyloid-like granular aggregates [27]. It appears to provide a new mechanism for triggers of tauopathies in the formaldehyde toxicosis.

## Materials and Methods

### Expression and purification of recombinant htau-40

The clone of recombinant human tau-40 was kindly provided by Dr. Goedert (University of Cambridge, UK) [9]. Protein tau, overexpressed in *E. coli*, was purified as described previously [52], [53]. The bacterial cells were homogenized with a sonicator, boiled at 100°C for 15 min and the protein was purified using Sepharose-Q, Sepharose-SP and Sephadex-50 chromatography columns (Pharmacia, USA). The concentration of recombinant tau was determined with a spectrophotometer (Hitachi U-2010, Japan) by measuring the absorbance at 280 nm [54]. Tau-40 appeared as a single band in SDS-PAGE after purification. Assay of tau was performed in the promotion of tubulin assembly following the Alonso's method [8]. Porcine brain tubulin was purified as described previously [27]. The specific activity of tau was the same as that described by Goedert [1].

### Tau aggregation in the presence of aldehyde

Neuronal tau with additions of different concentrations of formaldehyde (Sigma, USA) or acetaldehyde (ACROS Organic, USA) in 50 mM phosphate buffer (pH 7.2) at final tau concentration of 10–20 µM was incubated at 37°C overnight to allow the reaction to reach completion. The concentration of aldehyde-aggregated tau used was 10–20 µM according to the report that endogenous tau is present at 5–10 µM in human brain [55]. Then protein solution was diluted to the desired concentration using the phosphate buffer and 3 µl of the sample was deposited onto a mica surface and kept for 5 min at room temperature. The mica was rinsed with ultra-purified water 20 times and thoroughly dried with nitrogen gas before being observed by AFM. Glutaraldehyde (ICN Biomedicals Inc, USA) and BSA (Sigma, USA) were used as controls. For time-lapse analyses, aliquots were taken at different time intervals while tau was incubated with formaldehyde or acetaldehyde. Then the sample was observed under AFM or loaded onto 10% SDS-PAGE. To test whether tau polymerization is related to formaldehyde polymerization, 10% methanol was added when the tau protein was incubated in the presence of 0.1% formaldehyde at 37°C overnight. AFM studies were conducted on a Mutiplemode AFM with a NanoScope IIIa controller (Veeco Instruments, USA).

### DTNB and OPT modification

Neuronal tau was resuspended in 100 mM phosphate buffer containing OPT (Sigma, USA) or DTNB (Sigma, USA) at a molar ratio of 20/1 (reagent/protein) in the presence of formaldehyde at different concentrations at 37°C for 120 min. The absorbance (412 nm for DTNB modification) was then measured on a spectrophotometer (Hitachi U-2010, Japan). For OPT modification, the fluorescence (Ex 340 nm/Em 455 nm) was measured (slit = 5.0 nm for both Ex and Em) on a fluorescence spectrophotometer (Hitachi F-4500, Japan). Under the same conditions, tau was resuspended in 0.005% formaldehyde and 2 µM DTNB or 2 µM OPT and aliquots were taken to measure the absorbance and the fluorescence at different time intervals. The data were analyzed following Tsou's method [29].

### α-Synuclein and RNase A aggregation in the presence of formaldehyde

The clone of human α-Synuclein (as a gift from Prof. Hong-Yu Hu in the Institute of Biochemistry and Cell Biology, CAS, Shanghai) was expressed in *E. coli* and purified as described [56]. The purified α-synuclein showed a single protein band on SDS-PAGE. α-Synuclein was incubated in 100 mM phosphate buffer (pH 7.2) containing formaldehyde at different concentrations at 37°C over night, and then aliquots were taken for SDS-PAGE. RNase A (Sigma, USA) was pretreated with DTT (10 mM) in 100 mM phosphate buffer (pH 7.2) at 37°C for 1 h and dialysed at 4°C over night as described [31]. The dialysed RNase A was incubated with formaldehyde at different concentrations at 37°C for 4 h, and aliquots were then taken for SDS-PAGE. RNase A incubated without DTT was used as a control.

### Detection of tau aggregation in cells by thioflavin S

SY5Y cells were treated by 0.0005% formaldehyde for 72 h. Then, the cells on the coverslips were fixed with 4% paraformaldehyde in PBS for 15 min, permeabilized with 80% MeOH for 6 min at −20°C, incubated with 0.1% ThS (Sigma, USA) for 5 min, and washed three times in ethanol (50%). The samples were incubated with antibody Tau-1 (Chemicon, USA) in 5% goat serum PBS solution. The secondary anti-mouse antibody labeled with TR (Santa Cruz, USA) was also diluted with 5% goat serum in PBS and incubated for 45 min. The cells were washed twice with PBS and once with double-distilled water, and then mounted with Mowiol media containing DAPI. Cells containing distinct ThS signals to indicate the presence of the aggregated material with β-pleated sheets were scored in independent fields containing a total of 300 cells [33]. The fluorescence intensity of tau aggregation stained by ThS was analyzed with Lsmix software (Zeiss, Germany).

### Tau transfection and aggregation in the HEK 293 cells

HEK 293 cells were placed on 6-well tissue plates and transiently transfected with the appropriate HA-tau (as a gift from Dr. Ya-Jie Xu in the Institute of Biophysics, CAS) or control plasmid following the protocol described by the manufacturer. Typically, 1 µg of plasmid DNA and 4 µl of DMRIE-C reagent (Invitrogen, USA) were used per coverslip. The cells were incubated for 4 h in the transfection mixture prior to replacement with fresh culture medium. The transfected cells were visualized by fluorescence microscopy. After transfection for 24 h, HEK 293 cells were treated by 0.0002% formaldehyde for 72 h. Part of the cells was fixed for immunofluorescence, and the others were performed for immunoblotting [35]. The cells resuspended in NP-40 containing lysis buffer (10 mM Hepes, pH 7.4, 2 mM EGTA, 0.5% NP-40, 1 mM NaF, 1 mM NaVO_4_, 1 mM PMSF, 1 mM DTT, 50 mg/ml trypsin inhibitor, 10 mg/ml aprotinin and leupeptin) and placed on ice for 30 min. The lysates were centrifuged (12,000 g, 4°C, 12 min) and the protein concentration was determined. Equivalent samples (100 µg protein) were subjected to SDS-PAGE on 8% gel. The proteins were then transferred onto nitrocellulose membranes, and probed with anti-HA (Santa Cruz, USA) or anti β-actin antibodies (Sigma, USA) followed by the appropriate secondary antibodies conjugated to horseradish peroxidase (HRP) (KPL, Gaithersburg, Maryland, USA). Immunoreactive bands were visualized using enhanced chemiluminescence (Pierce, USA). The molecular sizes of the developed proteins were determined by comparison with prestained protein markers (Invitrogen, CA).

## Supporting Information

Figure S1 Formaldehyde-treated tau on SDS-PAGE. Conditions for the incubation of tau with formaldehyde were the same as those for Figure 1. Desired concentrations of formaldehyde were used as indicated. Protein tau solution with varied formaldehyde concentrations was used for each load in 10% SDS-PAGE (A). The gray densities of the protein bands on SDS-PAGE were measured (B) Curves 1 and 2 in B represent the densities of tau polymer and monomer, respectively. As a control, acetaldehyde of the same concentration range as that of formaldehyde under the same conditions was used (C), with the gray densities shown in D.
(0.44 MB EPS)

Figure S2 Neuronal tau incubated with different concentrations of acetaldehyde observed by AFM. Conditions were referred to Figure 1, except that the formaldehyde was replaced by acetaldehyde. The neuronal tau was incubated with acetaldehyde at 0.1% (A), 0.5% (B) and 1.0% (C) concentrations (Bar: 100 nm). The mean values of the horizontal diameters of observed tau particles at different acetaldehyde concentrations are presented in D.
(0.82 MB EPS)

Figure S3 Crosslinking of tau in glutaraldehyde solutions at different concentrations. Conditions are referred to Figure 1, except that the formaldehyde was replaced by glutaraldehyde as a positive control. The images are not shown here, but the mean values of the horizontal diameters of observed neuronal tau (Curve 1) and BSA (Curve 2) particles against the glutaraldehyde concentration are displayed.
(0.08 MB EPS)
